# Direct Endovascular Versus Bridging Therapy in M2 Segment Occlusion of Middle Cerebral Artery: A MR CLEAN Registry Study

**DOI:** 10.1161/STROKEAHA.125.051967

**Published:** 2025-07-22

**Authors:** Mohamed F. Doheim, Robrecht R.M.M. Knapen, Julie Staals, Wouter J. Schonewille, Diederik W.J. Dippel, Adriaan C.G.M. van Es, Hester F. Lingsma, Christiaan van der Leij, Charles B. Majoie, Raul G. Nogueira, Robert J. van Oostenbrugge, Wim H. van Zwam

**Affiliations:** Department of Neurology, Stroke Institute, University of Pittsburgh Medical Center, PA (M.F.D., R.G.N.).; Departments of Radiology and Nuclear Medicine (R.R.M.M.K., C.v.d.L., W.H.v.Z.), Maastricht University Medical Center+, the Netherlands.; Department of Neurology (R.R.M.M.K., J.S., R.J.v.O., M.F.D.), Maastricht University Medical Center+, the Netherlands.; School for Cardiovascular Diseases Maastricht (CARIM), Maastricht University, the Netherlands (R.R.M.M.K., R.J.v.O., W.H.v.Z.).; Research Institute for Oncology and Reproduction (GROW), School for Oncology and Reproduction (C.v.d.L.), Maastricht University, the Netherlands.; Department of Radiology and Nuclear Medicine, Maastricht University Medical Center, the Netherlands (R.R.M.M.K., C.v.d.L., W.H.v.Z.).; Department of Neurology, Maastricht University Medical Center, the Netherlands (R.J.v.O.).; Department of Neurology, St. Antonius Hospital, Nieuwegein, the Netherlands (W.J.S.).; Departments of Neurology (D.W.J.D.), Erasmus University Medical Center, Rotterdam, the Netherlands.; Departments of Public Health (H.F.L.), Erasmus University Medical Center, Rotterdam, the Netherlands.; Department of Radiology and Nuclear Medicine, Leiden University Medical Center, the Netherlands (A.C.G.M.v.E.).; Department of Radiology and Nuclear Medicine, Amsterdam University Medical Centers, University of Amsterdam, the Netherlands (C.B.M.).; (Department of Radiology, Erasmus University Medical Center); (Department of Neurology, Amsterdam UMC, University of Amsterdam, Amsterdam); (Department of Neurology, Haaglanden MC, the Hague); (Department of Radiology, St. Antonius Hospital, Nieuwegein); (Department of Radiology and Nuclear Medicine, Amsterdam UMC, University of Amsterdam); (Departments of Neurology and Radiology, Erasmus University Medical Center); (Department of Neurology, and Department of Radiology, Maastricht University Medical Center and Cardiovascular Research Institute Maastricht (CARIM); (Department of Radiology, Erasmus University Medical Center); (Department of Radiology and Nuclear Medicine, Amsterdam UMC, University of Amsterdam, Amsterdam); (Department of Neurology, Amsterdam UMC, University of Amsterdam, Amsterdam Sanne J. den Hartog (Departments of Neurology, Radiology, and Public Health, Erasmus University Medical Center); (Departments of Neurology and Radiology, Maastricht University Medical Center and Cardiovascular Research Institute Maastricht (CARIM); (Department of Neurology, Erasmus University Medical Center); (Department of Radiology, Erasmus University Medical Center); (Department of Neurology, Amsterdam UMC, University of Amsterdam, Amsterdam); (Department of Radiology and Nuclear Medicine, Amsterdam UMC, University of Amsterdam, Amsterdam); (Department of Neurology, Amsterdam UMC, University of Amsterdam, Amsterdam); (Department of Radiology, St. Antonius Hospital, Nieuwegein); (Department of Neurology, Leiden University Medical Center); (Department of Radiology, Leiden University Medical Center); (Department of Neurology, Rijnstate Hospital, Arnhem); (Department of Radiology, Rijnstate Hospital, Arnhem); (Department of Radiology, Haaglanden MC, the Hague); (Department of Neurology, Haaglanden MC, the Hague); (Department of Neurology, HAGA Hospital, the Hague); (Department of Radiology, HAGA Hospital, the Hague); (Department of Neurology, University Medical Center Utrecht); (Department of Radiology, University Medical Center Utrecht); (Department of Neurology, Radboud University Medical Center, Nijmegen); (Department of Neurosurgery, Radboud University Medical Center, Nijmegen); (Department of Neurology, Isala Klinieken, Zwolle); (Department of Neurology, Elisabeth-TweeSteden ziekenhuis, Tilburg); (Department of Neurology, Elisabeth-TweeSteden ziekenhuis, Tilburg); (Department of Radiology, Elisabeth-TweeSteden ziekenhuis, Tilburg); (Department of Neurology, Isala Klinieken, Zwolle); (Department of Neurology, Isala Klinieken, Zwolle); (Department of Radiology, Isala Klinieken, Zwolle); (Department of Neurology, Reinier de Graaf Gasthuis, Delft); (Department of Radiology, Reinier de Graaf Gasthuis, Delft); (Department of Neurology, University Medical Center Groningen); (Department of Radiology, University Medical Center Groningen); (Department of Radiology, University Medical Center Groningen); (Department of Neurology, Atrium Medical Center, Heerlen); (Department of Radiology, Atrium Medical Center, Heerlen); (Department of Neurology, Catharina Hospital, Eindhoven); (Department of Radiology, Catharina Hospital, Eindhoven); (Department of Neurology, Isala Klinieken, Zwolle); (Department of Neurology, Medisch Spectrum Twente, Enschede); (Department of Neurology, Medisch Spectrum Twente, Enschede); (Department of Radiology, Erasmus University Medical Center); (Department of Radiology, Haaglanden MC, the Hague); (Department of Radiology, Leiden University Medical Center); (Department of Radiology and Nuclear Medicine, Amsterdam UMC, University of Amsterdam); (Department of Radiology, Radboud University Medical Center, Nijmegen); (Department of Radiology and Nuclear Medicine, Amsterdam UMC, University of Amsterdam); (Department of Radiology, Texas Stroke Institute); (Department of Radiology and Nuclear Medicine, Amsterdam UMC, University of Amsterdam); (Department of Radiology, Maastricht University Medical Center and Cardiovascular Research Institute Maastricht [CARIM]); (Department of Radiology and Nuclear Medicine, Amsterdam UMC, University of Amsterdam); (Department of Radiology, Haaglanden MC, the Hague); (Department of Radiology, Haaglanden MC, the Hague); (Department of Radiology and Nuclear Medicine, Amsterdam UMC, University of Amsterdam); (Department of Radiology, Rijnstate Hospital, Arnhem); (Department of Radiology, Catharina Hospital, Eindhoven); (Department of Radiology, St. Antonius Hospital, Nieuwegein); (Department of Radiology, Amsterdam UMC, Vrije Universiteit van Amsterdam, Amsterdam); (Department of Radiology, Erasmus University Medical Center); (Department of Radiology, Radboud University Medical Center, Nijmegen); (Department of Radiology, Haaglanden MC, the Hague); (Department of Radiology, University Medical Center Groningen); (Department of Radiology, Noordwest Ziekenhuisgroep, Alkmaar); (Department of Radiology, Catharina Hospital, Eindhoven); (Department of Radiology, Elisabeth-TweeSteden ziekenhuis, Tilburg); (Department of Neurosurgery, Radboud University Medical Center, Nijmegen); (Department of Radiology, University Medical Center Utrecht); (Department of Radiology, Albert Schweitzer Hospital, Dordrecht); (Department of Radiology, University Medical Center Groningen); (Department of Radiology, HAGA Hospital, the Hague); (Department of Neurology Radiology, Radboud University Medical Center, Nijmegen); (Department of Radiology, University Medical Center Groningen); (Department of Neurosurgery, Radboud University Medical Center, Nijmegen); (Department of Radiology, Erasmus University Medical Center); (Department of Radiology, Maastricht University Medical Center and Cardiovascular Research Institute Maastricht [CARIM]); (Department of Radiology, Erasmus University Medical Center); (Department of Neurology, Amsterdam UMC, University of Amsterdam); (Department of Radiology, Haaglanden MC, the Hague); (Department of Neurology, Haaglanden MC, the Hague); (Department of Radiology, St. Antonius Hospital, Nieuwegein); (Department of Neurology, Rijnstate Hospital, Arnhem); (Department of Radiology, Rijnstate Hospital, Arnhem); (Department of Neurology, University Medical Center Utrecht); (Department of Radiology, University Medical Center Utrecht); (Department of Radiology, Rijnstate Hospital, Arnhem); (Department of Radiology, Isala Klinieken, Zwolle); (Department of Neurology, Erasmus University Medical Center); (Department of Neurology, Erasmus University Medical Center); (Department of Neurology, St. Antonius Hospital, Nieuwegein); (Department of Neurology, Amsterdam UMC, University of Amsterdam); (Department of Neurology, Rijnstate Hospital, Arnhem); (Department of Neurology, Rijnstate Hospital, Arnhem); (Department of Radiology, Rijnstate Hospital, Arnhem); (Department of Neurology, Haaglanden MC, the Hague); (Department of Neurology, Haaglanden MC, the Hague); (Department of Neurology, Radboud University Medical Center, Nijmegen); (Department of Neurology, Isala Klinieken, Zwolle); (Department of Neurology, Atrium Medical Center, Heerlen); (Department of Neurology, University Medical Center Groningen); (Department of Neurology, University Medical Center Groningen); (Department of Neurology, Catharina Hospital, Eindhoven); Department of Neurology, Reinier de Graaf Gasthuis; (Department of Neurology, Medisch Spectrum Twente, Enschede); (Department of Neurology, Medisch Spectrum Twente, Enschede); (Department of Neurology, Medisch Spectrum Twente, Enschede); (Department of Neurology, Maastricht University Medical Center and Cardiovascular Research Institute Maastricht [CARIM]); (Department of Neurology, Maastricht University Medical Center and Cardiovascular Research Institute Maastricht [CARIM]); (Department of Neurology, Maastricht University Medical Center and Cardiovascular Research Institute Maastricht [CARIM]); (Department of Neurology, Leiden University Medical Center); (Department of Neurology, HAGA Hospital, the Hague); (Department of Neurology, HAGA Hospital, the Hague); (Department of Neurology, Rijnstate Hospital, Arnhem); (Department of Neurology, University Medical Center Utrecht); (Department of Neurology, Elisabeth-TweeSteden ziekenhuis, Tilburg); (Department of Neurology, Isala Klinieken, Zwolle); (Department of Neurology, Radboud University Medical Center, Nijmegen); (Department of Neurology, Radboud University Medical Center, Nijmegen); (Department of Radiology, Leiden University Medical Center); (Department of Public Health, Erasmus University Medical Center); (Departments of Neurology and Public Health, Erasmus University Medical Center); (Department of Radiology and Nuclear Medicine, Amsterdam UMC, University of Amsterdam); (Department of Neurology, Radboud University Medical Center, Nijmegen); (Department of Neurology, Erasmus University Medical Center); (Department of Neurology, Erasmus University Medical Center); (Department of Neurology, Erasmus University Medical Center); (Department of Neurology, Erasmus University Medical Center); (Department of Neurology, Erasmus University Medical Center; Department of Radiology and Nuclear Medicine, Amsterdam UMC, University of Amsterdam; Department of Radiology, Maastricht University Medical Center and Cardiovascular Research Institute Maastricht [CARIM]); (Department of Radiology and Nuclear Medicine, Amsterdam UMC, University of Amsterdam); (Department of Radiology and Nuclear Medicine, Amsterdam UMC, University of Amsterdam); (Department of Radiology and Nuclear Medicine, Amsterdam UMC, University of Amsterdam); (Department of Radiology and Nuclear Medicine, Amsterdam UMC, University of Amsterdam); (Department of Radiology and Nuclear Medicine, Amsterdam UMC, University of Amsterdam); (Department of Radiology and Nuclear Medicine, Amsterdam UMC, University of Amsterdam); (Department of Radiology and Nuclear Medicine, Biomedical Engineering & Physics, Amsterdam UMC, University of Amsterdam); (Department of Radiology and Nuclear Medicine, Amsterdam UMC, University of Amsterdam); (Department of Radiology and Nuclear Medicine, Amsterdam UMC, University of Amsterdam); (Department of Radiology and Nuclear Medicine, Amsterdam UMC, University of Amsterdam); (Department of Neurology, Rijnstate Hospital, Arnhem); (Department of Radiology and Nuclear Medicine, Amsterdam UMC, University of Amsterdam); (Departments of Neurology and Radiology, University Medical Center Groningen); (Department of Neurology, Amsterdam UMC, University of Amsterdam, Amsterdam); (Department of Neurology, Amsterdam UMC, University of Amsterdam, Amsterdam); (Department of Biomedical Engineering & Physics, Amsterdam UMC, University of Amsterdam); (Department of Biomedical Engineering & Physics, Amsterdam UMC, University of Amsterdam); (Department of Biomedical Engineering & Physics, Amsterdam UMC, University of Amsterdam); (Department of Biomedical Engineering & Physics, Amsterdam UMC, University of Amsterdam); (Departments of Neurology, Radiology, and Public Health, Erasmus University Medical Center); (Department of Neurology, Maastricht University Medical Center and Cardiovascular Research Institute Maastricht [CARIM]); (Department of Radiology and Nuclear Medicine, Amsterdam UMC, University of Amsterdam)

**Keywords:** intracranial hemorrhage, ischemic stroke, middle cerebral artery, mortality

## Abstract

**BACKGROUND:**

The optimal strategy for managing M2 segment occlusions of the middle cerebral artery, whether with direct endovascular treatment (EVT) or bridging therapy with intravenous thrombolysis (IVT) before EVT, remains unclear. This study aimed to evaluate the effectiveness and safety of both approaches.

**METHODS:**

Patients with M2 segment occlusions of the middle cerebral artery, treated between March 2014 and December 2018, were identified from the MR CLEAN Registry (Multicenter Randomized Clinical Trial of Endovascular Treatment for Acute Ischemic Stroke in the Netherlands), a prospective, nationwide, multicenter registry of patients with acute ischemic stroke who underwent endovascular treatment during that period. They were divided into 2 groups: those who received IVT followed by EVT, and those who received EVT alone. Primary outcomes included functional outcomes at 90 days, assessed by ordinal logistic regression analysis of modified Rankin Scale (mRS) scores. Secondary outcomes included recanalization rates measured by extended Thrombolysis in Cerebral Infarction scores, dichotomized mRS scores (0–1, 0–2, and 0–3), death at 90 days, and symptomatic intracranial hemorrhage. All analyses were performed using both unadjusted and adjusted multivariable approaches, with adjustment achieved through inverse probability of treatment weighting to account for baseline imbalances, including age, baseline National Institutes of Health Stroke Scale score, prior stroke, history of atrial fibrillation, anticoagulant use, and transfer status.

**RESULTS:**

A total of 539 patients with M2 occlusions were included in the analysis: 377 received IVT+EVT and 162 received EVT alone. The median age was significantly lower in the IVT+EVT group compared with the EVT-alone group (71 [61–79] versus 74 [65–81]; *P*=0.01), whereas the proportion of male patients was similar between groups (55.2% versus 51.9%; *P*=0.15). At 90 days, inverse probability of treatment weighting analysis showed that IVT+EVT was significantly associated with reduced disability compared with EVT alone (adjusted common odds ratio for mRS score, 1.52 [95% CI, 1.04–2.21]; *P*=0.03). Dichotomized functional outcomes and mortality were numerically in favor of IVT+EVT, with higher rates of mRS score of 0 to 1 (38.9% versus 29.7%, aOR, 1.40 [95% CI, 0.85–2.30]; *P*=0.19), mRS score of 0 to 2 (57.8% versus 46.5%; aOR, 1.42 [95% CI, 0.88–2.29]; *P*=0.15), and mRS score of 0 to 3 (73.2% versus 59.4%, aOR, 1.54 [95% CI, 0.94–2.51]; *P*=0.09), as well as lower 90-day mortality (17.2% versus 25.8%; aOR, 0.83 [95% CI, 0.47–1.45]; *P*=0.51). In contrast, recanalization rates and symptomatic intracranial hemorrhage were numerically in favor of EVT alone, but all these differences were not statistically significant (*P*>0.05).

**CONCLUSIONS:**

Bridging therapy may yield superior functional outcomes compared with EVT alone for patients with the middle cerebral artery–M2 occlusions.

Endovascular treatment (EVT) is the standard of care for eligible patients with acute ischemic stroke with proximal large vessel occlusions in the anterior circulation, with proven efficacy extending up to 24 hours from symptom onset.^1–3^ However, trials focused on patients with middle cerebral artery (MCA)–M1 and intracranial internal carotid artery occlusions.^1–3^ Previous studies highlight poor outcomes for untreated MCA-M2 occlusions (45.8%), with relatively high 6-month mortality rates (20.8%).^4^ A multicenter study similarly reported high morbidity and mortality for MCA-M2 occlusions, with 60% disability and 24% mortality at 6 months.^5^ Although the DISTAL (Endovascular Treatment for Stroke due to Occlusion of Medium or Distal Vessels) and ESCAPE-MeVO (Endovascular Treatment of Stroke due to Medium-Vessel Occlusion) trials found no significant reduction in disability or mortality with EVT compared with best medical treatment for medium and distal vessel occlusions, their focus on primarily distal and nondominant occlusions suggests that EVT remains indicated for dominant and proximal M2 occlusions.^6,7^ Likewise, the American Heart Association/American Stroke Association guidelines endorse treatment with stent retrievers as a reasonable option within 6 hours of symptom onset (class IIb).^8^ Moreover, the European Stroke Organization guidelines reflect a unanimous consensus among the guideline committee that patients with proximal M2 occlusions met the inclusion criteria for most randomized trials, supporting the reasonableness of EVT in these cases.^9^ Evidence from the HERMES (Highly Effective Reperfusion Evaluated in Multiple Endovascular Stroke Trials) patient-level data subgroup analysis of 5 randomized controlled trials (RCTs), along with results from large registries, demonstrates favorable EVT outcomes for predominantly proximal M2 occlusions in both early and late treatment windows.^10–12^

The optimal strategy for managing M2 segment of MCA occlusions, whether direct EVT or bridging therapy with intravenous thrombolysis (IVT) plus EVT, remains unclear. The IRIS (Value of Intravenous Thrombolysis in Endovascular Treatment for Large-Vessel Anterior Circulation Stroke) individual participant data meta-analysis evaluated the noninferiority of EVT alone versus IVT plus EVT in patients presenting directly to EVT-capable centers. Although noninferiority was not established, the study emphasized the need for further research on cost-effectiveness and personalized treatment strategies. The differences in functional outcomes between the 2 treatment groups were minimal and statistically insignificant.^13^ However, IRIS study findings may lack generalizability to M2 occlusions, as no subgroup analysis was conducted due to the limited number of M2 cases among the 6 included trials.^13–19^ Subgroup analyses from MR CLEAN No IV (A Randomized Trial of Intravenous Alteplase Before Endovascular Treatment for Stroke), DIRECT-MT (Endovascular Thrombectomy With or Without Intravenous Alteplase in Acute Stroke), and DEVT (Effect of Endovascular Treatment Alone vs Intravenous Alteplase Plus Endovascular Treatment on Functional Independence in Patients With Acute Ischemic Stroke) trials limited by sample size did not show an interaction effect, nor a trend towards different effects in M2.^14,15,17^

IVT may be more beneficial for medium or distal occlusions, especially in transfer patients, as these tend to have a lower thrombus load compared with large vessel occlusions, and IVT may result in partial to complete recanalization.^20,21^ Real-world data from the MR CLEAN Registry provide a valuable opportunity to examine these treatment strategies across diverse clinical settings. Because the current guidelines recommend IVT administration when no contraindications exist, patients in the EVT-alone group may inherently have contraindications to IVT. Therefore, a meaningful analysis requires a statistical method that minimizes this potential bias. This study aims to compare the efficacy and safety of IVT followed by EVT versus EVT alone in patients with M2 MCA occlusions. In addition, it will assess the influence of prehospital pathways, examining outcomes based on whether patients were directly admitted to a comprehensive stroke center (mothership) or transferred from a primary stroke center (drip-and-ship).

## Methods

The corresponding author had full access to all study data and assumes responsibility for its accuracy and analysis. Due to legal restrictions regarding patient privacy, source data cannot be shared. However, detailed statistical analyses and methodologies are available upon reasonable request to the corresponding author. This study adhered to the Strengthening the Reporting of Observational Studies in Epidemiology guidelines.

### Study Design and Population

This study analyzed data from the MR CLEAN Registry (Multicenter Randomized Clinical Trial of Endovascular Treatment for Acute Ischemic Stroke in the Netherlands), collected between March 2014 and December 2018. The MR CLEAN Registry is a prospective, nationwide, observational cohort study conducted across all Dutch interventional stroke centers, capturing data on all patients treated with EVT for acute ischemic stroke since the conclusion of the MR CLEAN trial in March 2014. The study, whose design and methodology have been detailed in prior publications, spans 3 phases: part I (March 2014–June 15, 2016), part II (June 16, 2016–November 1, 2017), and part III (November 2, 2017–December 31, 2018).^22–24^ The MR CLEAN Registry protocol was reviewed and approved by the ethics committee of Erasmus University Medical Center in Rotterdam (MEC-2014-235) and subsequently received approval from the research boards of all participating centers.

The analysis included patients aged ≥18 years with MCA-M2 occlusions, as identified on baseline angiographic imaging, including those with tandem occlusions. Notably, prior evaluations of this registry indicated that 71% of recorded MCA-M2 cases involved proximal M2 segments.^25^ To ensure homogeneity and minimize bias, patients with other distal medium vessel occlusions—such as MCA-M3, anterior cerebral artery, or posterior circulation occlusions—and those with significant prestroke disability (modified Rankin Scale [mRS] score >2) were excluded. Patients were categorized into 2 treatment groups: bridging therapy (IVT followed by EVT) and EVT alone, which included those with contraindications to IVT. Among mothership patients, IVT administration could overlap with the initiation of EVT. EVT involved arterial puncture, catheter navigation, and thrombus removal using stent retrievers, aspiration techniques, or a combination of both, with the choice of technique and device left to the discretion of the treating interventionist. Across the MR CLEAN Registry, the use of direct aspiration increased from 13% in both 2014 and 2015% to 27% in 2016, peaking at 37% in 2017 and remaining steady at 36% in 2018. The MCA-M2 became more common in later years, increasing from 9% in 2014% to 26% in 2018.^24^

### Data Variables

Data collected for this study included patient age, sex, baseline National Institutes of Health Stroke Scale (NIHSS) score, and prestroke mRS score. Medical history variables encompassed prior stroke, atrial fibrillation, hypertension, hypercholesterolemia, and diabetes. In addition, collateral grading, Alberta Stroke Program Early CT Score, and transfer status from a primary stroke center were recorded. Procedural variables included stroke symptom onset-to-groin time and procedure duration and stroke symptom onset-to-recanalization time. An independent core laboratory, blinded to clinical data except for the symptom side, reviewed baseline non-contrast computed tomography (NCCT) and computed tomography angiography, procedural digital subtraction angiography, and follow-up NCCT in cases of suspected hemorrhage or clinical decline. Assessments included Alberta Stroke Program Early CT Score, occlusion location, and collateral grading, based on baseline NCCT or computed tomography angiography, using standardized instructions and definitions.^26,27^

### Outcomes

The primary outcome included reduced disability on the mRS score at 90 days, which is a 7-point ordinal scale ranging from 0 no symptoms to 6 dead. The mRS score was assessed by trained research nurses through either telephone or in-person interviews. Secondary outcomes included neurological deficit at 24 to 48 hours after stroke onset, assessed by NIHSS scores and binary mRS scores, categorized as excellent outcomes (mRS score of 0–1), functional independence or good outcomes (mRS score of 0–2), and fair outcomes (mRS score of 0–3). Procedural outcomes included stroke symptom onset-to-groin time, procedure duration, and recanalization success based on an extended Thrombolysis in Cerebral Infarction (eTICI) grades.^28^ Successful recanalization was defined as eTICI 2b-3, excellent recanalization as eTICI 2c/3, and complete recanalization as eTICI 3. Safety outcomes comprised 90-day mortality and periprocedural symptomatic intracranial hemorrhage (sICH). sICH was defined as neurological worsening, characterized by an increase of ≥4 points on the NIHSS, accompanied by evidence of related ICH on follow-up imaging, either NCCT or magnetic resonance imaging. Follow-up imaging was evaluated by a central imaging core laboratory, which determined the presence and location of ICH according to the Heidelberg Bleeding Classification.^29^ Core laboratory members also evaluated procedural complications, such as distal emboli and new thrombi in separate vascular territories, using digital subtraction angiography.

### Statistical Analysis

All analyses were performed using Stata (StataCorp LLC, College Station, TX) and R (R Foundation for Statistical Computing, Vienna, Austria). Baseline characteristics were compared using χ^2^ or Fisher exact tests for categorical variables and Mann-Whitney *U* tests for continuous variables. Multivariable logistic regression models assessed the relationship between treatment approach and outcomes, adjusting for baseline imbalances including age, NIHSS score, previous stroke, history of atrial fibrillation, anticoagulation, and transfer status. Ordinal logistic regression analysis of mRS scores at 90 days provided adjusted common odds ratios (ORs). To reduce confounding, inverse probability treatment weighting (IPTW) was applied using propensity scores estimated from logistic regression, accounting for imbalances in baseline characteristics. These weights were incorporated into multivariable logistic regression, with robust standard errors to adjust for variance. Age and NIHSS score were included as prespecified adjustment variables due to their known association with clinical outcomes. Additional covariates were selected based on baseline imbalances identified in univariate analysis (*P*≤0.05), allowing for thorough adjustment for potential confounding factors.^30,31^ Subgroup analyses compared outcomes between transfer patients (initially treated at a primary stroke center) and mothership patients (directly admitted to an EVT-capable center). We also examined the interaction between treatment and transfer status, along with presenting outside business hours and first-line technique (aspiration versus stent retriever or combined technique) on the primary outcome (mRS score reduction at 90 days) using a multiplicative interaction term. We addressed missing data using complete case analysis to maintain transparency and avoid the assumptions of imputation methods.^32,33^ Statistical significance was defined as *P*<0.05.

### Role of Funding Source

The Applied Scientific Institute for Neuromodulation (Toegepast Wetenschappelijk Instituut voor Neuromodulatie) had no involvement in the trial design, patient enrollment, data collection, analysis, or preparation of the manuscript.

## Results

### Baseline and Clinical Characteristics

**Figure 1.**
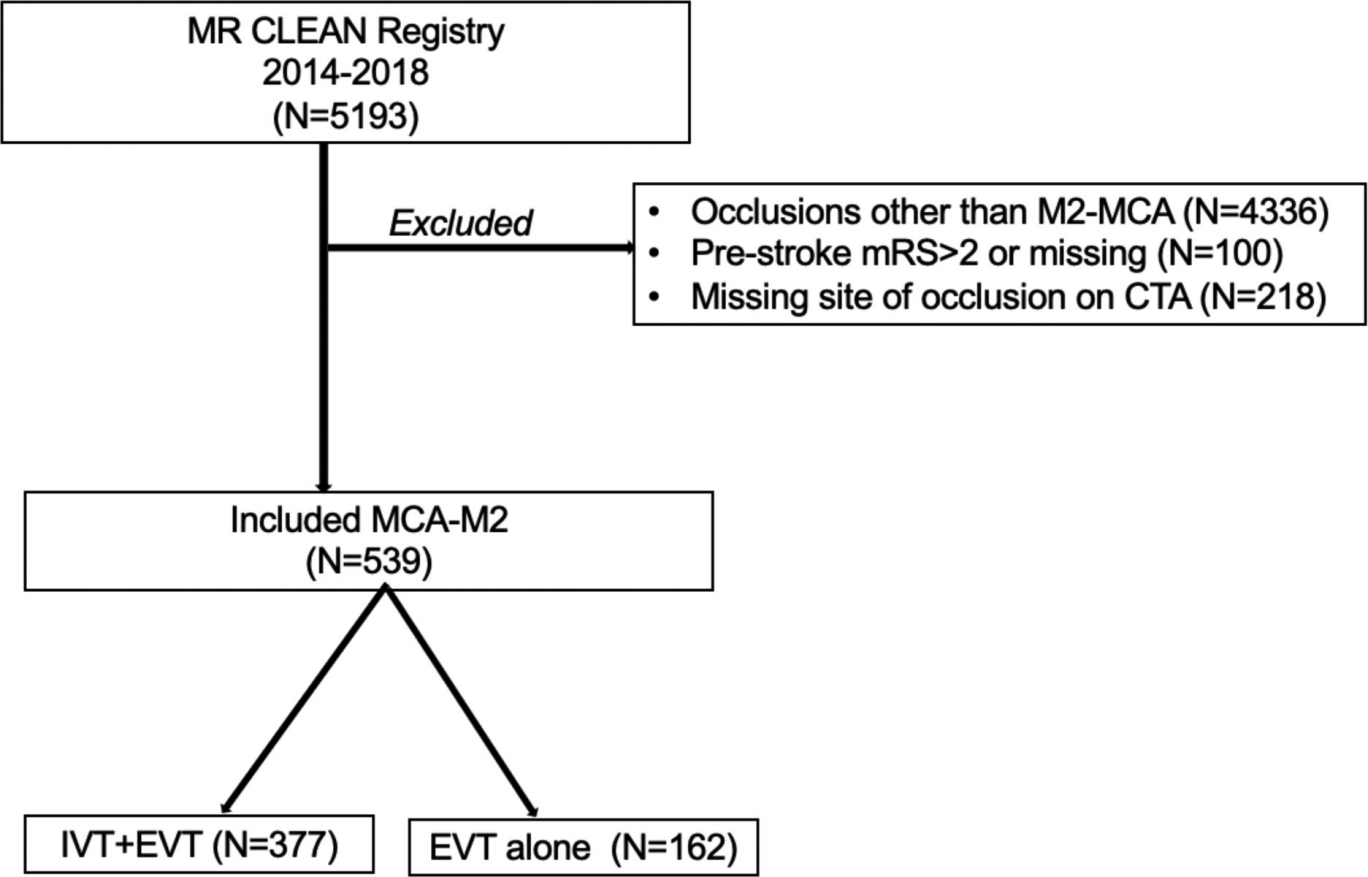
**Flowchart of included patients.** CTA indicates computed tomography angiography; EVT, endovascular treatment; IVT, intravenous thrombolytic; MCA, middle cerebral artery; MR CLEAN, Multicenter Randomized Clinical Trial of Endovascular Treatment for Acute Ischemic Stroke in the Netherlands; and mRS, modified Rankin Scale.

Among 5193 patients in the MR CLEAN Registry, 539 met the eligibility criteria for this study, with 377 in the IVT+EVT group and 162 in the EVT-alone group (Figure 1). The median age was slightly lower in the IVT+EVT group compared with the EVT-alone group (71 years [interquartile range (IQR), 61–79] versus 74 years [IQR, 65–81]; *P*=0.01). The proportion of males was similar in both groups (55.2% versus 51.9%; *P*=0.15). The median NIHSS-baseline scores were comparable between the groups (10 [IQR, 7.0–16.0] versus 11 [IQR, 6.0–18.0], *P*=0.66). A higher percentage of patients in the IVT+EVT group had a pre-mRS score of 0 to 1 (90.5% versus 86.4%; *P*=0.17). Previous stroke was less common in the IVT+EVT group compared with the EVT-alone group (14.2% versus 24.2%; *P*=0.005). Atrial fibrillation was significantly lower in the IVT+EVT group (19.0% versus 39.0%; *P*<0.001). Anticoagulation use was also lower in the IVT+EVT group (1.9% versus 16.9%; *P*<0.001). In addition, transfer from a primary stroke center was more frequent in the IVT+EVT group (48.2% versus 23.5%, *P*<0.001; Table 1).

**Table 1.**
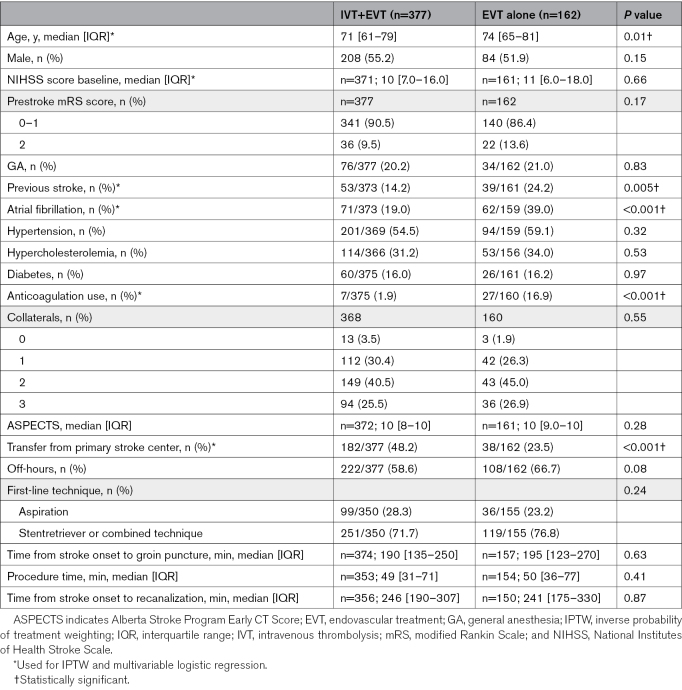
Baseline Patient Characteristics

### Clinical Outcomes

**Figure 2.**
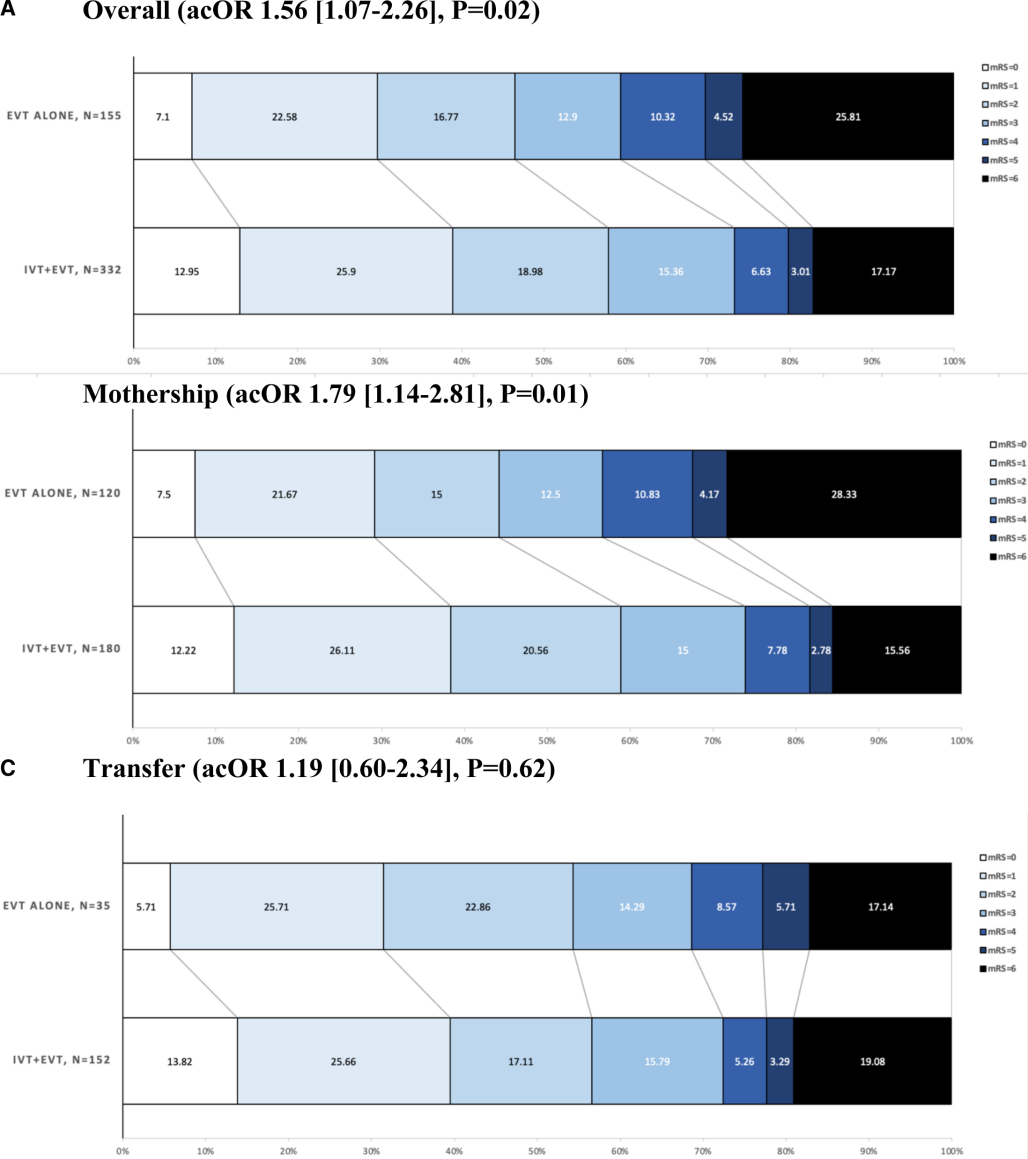
**Distribution of modified Rankin Scale (mRS) scores at 90 days comparing outcomes between patients treated with intravenous thrombolysis (IVT) plus endovascular treatment (EVT) and those treated with EVT alone. A**, Overall population. **B**, Mothership patients. **C**, Transfer patients. acOR indicates adjusted common odds ratio.

At 90 days, for mRS score (ordinal shift analysis), the unadjusted analysis showed a median (IQR) of 2 (1–4) in the IVT+EVT group versus 3 (1–6) in the EVT-alone group (*P*=0.004; Table 2). At 90 days, IPTW analysis showed that IVT+EVT was significantly associated with reduced disability compared with EVT alone (adjusted common OR for mRS score, 1.52 [95% CI, 1.04–2.21]; *P*=0.03; Table 3). In addition, the IVT+EVT subgroup analysis demonstrated a more substantial benefit in reduced disability within the mothership group (adjusted common OR for mRS score shift, 1.79 [95% CI, 1.14–2.81]; *P*=0.01), whereas no significant effect was observed in the transfer group (adjusted common OR, 1.19 [95% CI, 0.60–2.34]; *P*=0.62) with *P*_interaction_ of 0.02 (Figure 2).

**Table 2.**
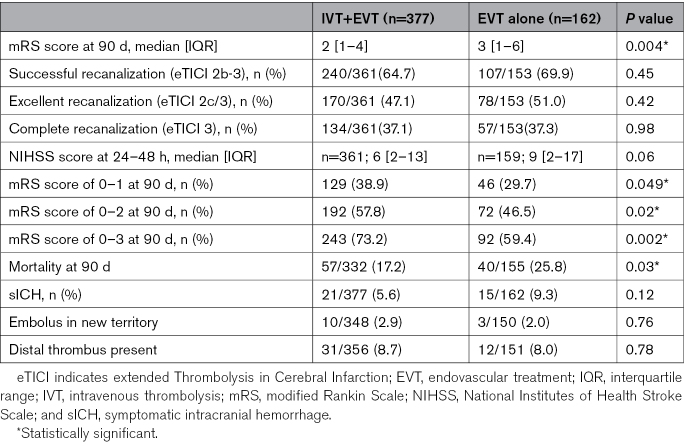
Outcomes Between Bridging (IVT+EVT) Versus EVT Alone in Overall Cohort

**Table 3.**
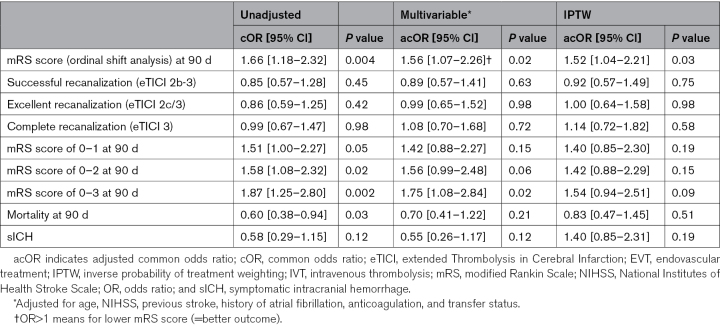
Outcomes in Adjusted Multivariable and IPTW Analysis of Bridging (IVT+EVT) Versus EVT Alone

The median NIHSS scores at 24 to 48 hours were numerically lower in the IVT+EVT group compared with the EVT-alone group (6 [IQR, 2–13] versus 9 [IQR, 2–17]; *P*=0.06). Dichotomized functional outcomes and mortality were numerically in favor of IVT+EVT, with higher rates of mRS score of 0 to 1 (38.9% versus 29.7%; adjusted OR [aOR], 1.40 [95% CI, 0.85–2.30]; *P*=0.19), mRS score of 0 to 2 (57.8% versus 46.5%, aOR, 1.42 [95% CI, 0.88–2.29]; *P*=0.15), and mRS score of 0 to 3 (73.2% versus 59.4%, aOR, 1.54 [95% CI, 0.94–2.51]; *P*=0.09; Tables 2 and 3).

To further explore outcome differences, we stratified mRS score distributions by thrombectomy technique: aspiration versus stent retriever or combined approaches. Within each subgroup, outcomes were further stratified by bridging IVT status. As summarized in Table S1, patients treated with bridging therapy consistently exhibited numerically higher rates of favorable outcomes (mRS score of 0–2) and lower mortality across both EVT technique groups. For instance, within the stent retriever/combined subgroup, the mRS score of 0 to 2 rate was 59.0% with bridging versus 47.0% without, and mortality was 17.1% versus 27.0%, respectively. Similar trends were observed in the aspiration subgroup (mRS score of 0–2: 55.7% with bridging versus 39.4% without; mortality: 19.3% versus 27.3%). Moreover, formal adjusted interaction analysis showed no statistically significant interaction (*P*_interaction_=0.74).

To investigate whether the effect of IVT bridging differs based on treatment timing, we conducted logistic regression models with an interaction term between bridging status and off-hours presentation. In the overall cohort, bridging therapy was associated with higher rates of favorable 90-day outcomes (mRS score of 0–2) compared with nonbridging during both off-hours (54.6% versus 46.6%) and business hours (62.3% versus 46.2%). Mortality was lower with bridging during off-hours (14.9% versus 26.2%) and slightly lower during business hours (20.3% versus 25.0%; Table S2). Among transferred patients, off-hour outcomes were similar between bridging and nonbridging groups in terms of mRS score of 0 to 2 (54.4% versus 55.6%) and mortality (16.7% versus 14.8%). During business hours, bridging patients had numerically better mRS score of 0 to 2 outcomes (59.7% versus 50.0%) but also slightly higher mortality (22.6% versus 25.0%). For mothership patients, bridging was associated with improved mRS score of 0 to 2 during off-hours (54.8% versus 43.4%) and business hours (64.5% versus 45.5%), with substantially lower mortality in off-hours (13.5% versus 30.3%) and modestly lower in business hours (18.4% versus 25.0%). Interaction analysis showed no statistically significant interaction between IVT bridging and off-hours presentation after adjustment for age and NIHSS score. This held for the overall cohort (*P*_interaction_=0.40), transferred patients (*P*_interaction_=0.54), and mothership patients (*P*_interaction_=0.60), indicating that the effect of bridging therapy was consistent regardless of treatment timing.

### Technical Outcomes

For successful recanalization (eTICI 2b-3), both unadjusted and adjusted analyses showed no statistically significant difference between the IVT+EVT and EVT-alone groups (64.7% [240/361] versus 69.9% [107/153], *P*=0.45; IPTW aOR, 0.92 [95% CI, 0.57–1.49]; *P*=0.75). Similarly, both excellent (eTICI 2C/3) and complete recanalization (eTICI 3) showed no statistically significant differences (Tables 2 and 3).

### Safety Outcomes

For periprocedural sICH, the unadjusted and adjusted analyses showed no statistically significant difference between the IVT+EVT and EVT-alone groups (5.6% [21/377] versus 9.3% [15/162], *P*=0.12; IPTW aOR, 1.40 [95% CI, 0.85–2.31]; *P*=0.19; Tables 2 and 3). For mortality at 90 days, the unadjusted analysis showed a significant difference between the IVT+EVT group and the EVT-alone group, but this difference was not statistically significant in the adjusted analysis (17.2% [57/332] versus 25.8% [40/155], *P*=0.03; IPTW aOR, 0.83 [95% CI, 0.47–1.45]; *P*=0.51; Tables 2 and 3).

## Discussion

The management of M2 segment occlusions MCA remains a subject of ongoing debate, with uncertainty surrounding whether direct EVT or bridging therapy (IVT followed by EVT) provides superior outcomes. Our analysis of the MR CLEAN Registry, which included 539 patients, offers valuable insights into the efficacy and safety of these treatment strategies, with subgroup analyses based on prehospital pathways (mothership versus transfer patients). Our findings demonstrate that IVT+EVT is associated with better functional outcomes compared with EVT alone, as evidenced by a significant improvement in functional outcomes for the IVT+EVT group. These results are consistent with previous studies suggesting that early partial or complete recanalization may be achieved with IVT, whereas it may enhance microvascular reperfusion, reduce infarct growth, and limit secondary ischemic injury, all of which contribute to improved outcomes.^14,34^ Although not supported by our results, IVT may help streamline the procedure by reducing catheterization attempts and shortening the overall procedural time.^35^ Moreover, the benefit of prior IVT itself is likely to be underestimated in any study comparing outcomes between IVT+EVT versus EVT alone, as patients with early recanalization after IVT and also those in whom there was a complete early neurological recovery without proven recanalization are excluded.

Several studies have demonstrated the safety and efficacy of EVT for MCA-M2 occlusions within the early time window. The HERMES subgroup analysis revealed that EVT for proximal M2 occlusions led to favorable outcomes, with 58.2% of patients achieving good functional outcomes (mRS score of 0–2).^10^ The STRATIS Registry (Systematic Evaluation of Patients Treated With Neurothrombectomy Devices for Acute Ischemic Stroke), comparing clinical and angiographic outcomes for M2 versus M1 occlusions, reported similar rates of successful reperfusion (91% for M1 versus 95% for M2), good functional outcomes (58% for M1 versus 59% for M2), and mortality (15% for M1 versus 14% for M2), although M2 occlusions had higher rates of sICH (4% versus 1%).^11^ Insights from the STAR Registry (Stroke Thrombectomy and Aneurysm Registry) in the 6- to 24-hour time window showed that M2 occlusions had similar rates of recanalization and good functional outcomes (43.9% versus 46.7%), and overall mortality was also similar (12.8% versus 13.9%).^12^ Untreated MCA-M2 occlusions were associated with poor outcomes, with 45.8% of patients experiencing unfavorable functional recovery (mRS score of ≥3) and a 6-month mortality rate of 20.8%, comparable to that of MCA-M1 occlusions.^4^ Another multicenter study revealed that MCA-M2 occlusions resulted in a high burden of morbidity and mortality, with 60% of patients experiencing significant disability and 24% succumbing to mortality within 6 months.^5^ Recently, 2 trials, DISTAL and ESCAPE-MeVO, investigated EVT for medium and distal vessel occlusions but found no significant reduction in disability or mortality compared with best medical treatment alone.^6,7^ DISTAL primarily included occlusions of the nondominant or codominant M2 segment of the MCA (129 [47.6%] in the EVT group versus 110 [40.4%] in the control group), along with other medium and distal vessel occlusions, including the M3 and M4 segments of the MCA, as well as the A1, A2, A3 (anterior cerebral artery) and P1, P2, P3 (posterior cerebral artery) segments.^7^ ESCAPE-MeVO, on the other hand, included several proximal M2 occlusions (64/253 [25.3%] in the EVT group versus 58/269 [21.6%] in the control group) and focused also on distal lesions, including the distal M2 and M3 segments of the MCA, A2, and A3 segments of the anterior cerebral artery, and P2 and P3 segments of the posterior cerebral artery, notably excluding A1 and P1 occlusions.^6^ Both trials reported a median NIHSS score of 6 to 8, suggesting the possible exclusion of more severe cases from enrollment and reflecting the generally mild to moderate severity of most medium vessel occlusions.^6,7^

The IRIS study demonstrated only a marginal benefit of IVT when combined with EVT in patients presenting directly to EVT-capable centers, with a mere 1.7% difference in mRS score of 0 to 2 at 90 days compared with EVT alone.^13^ This limited effect is modest relative to the substantial impact of EVT, which improves outcomes by ≈20%. The study also emphasized that delaying EVT for IVT administration may negate any potential benefit, as each hour of delay reduces functional independence by 6%. In contrast, our results, focused on M2 occlusions, showed a more pronounced difference: 38.9% versus 29.7% achieving an mRS score of 0 to 1 and 57.8% versus 46.5% achieving an mRS score of 0 to 2 in the IVT+EVT and EVT-alone groups, respectively. This suggests that IVT may provide greater benefit in M2 occlusions, potentially explaining the disparity between our findings and those of IRIS, highlighting the importance of tailoring treatment to occlusion site as shown in previous studies.^36–38^ A primary distinction between IRIS and this study is that IRIS exclusively included patients admitted directly to EVT-capable centers, and the time interval between IV thrombolysis administration and groin puncture/last angiography was considerably shorter, potentially limiting the time for IVT to exert its effect.^13^

We must consider that patients who responded to IVT and did not require EVT were excluded from the analysis, potentially introducing selection bias. This is particularly relevant for transfer patients, where excluding those who responded to IVT and did not need EVT left primarily IVT-refractory cases, which may have skewed the results.^21,39^ A meta-analysis, which included 18 studies and 7017 patients, demonstrated that the mothership model was superior to the drip-and-ship model for achieving functional independence at 90 days (OR, 1.34 [95% CI, 1.16–1.55]).^40^ However, no significant differences were observed between the 2 models in recanalization rates, sICH, or 90-day mortality, although longer onset-to-needle times were associated with worse outcomes.^40^ An important consideration in interpreting our findings is the potential influence of treatment timing, particularly presentation during on-hours versus off-hours. Prior studies have shown that off-hours presentation may be associated with workflow delays and variability in care delivery, which can impact outcomes.^41^ In our cohort, a higher proportion of patients in the EVT-alone group presented during off-hours compared with those who received IVT+EVT (66.7% versus 58.6%), although this difference was not statistically significant. Notably, patients in the IVT+EVT group had a significantly higher rate of interhospital transfer (48.2% versus 23.5%), suggesting that IVT was often initiated at the referring facility. This may have served as a temporizing intervention during transfer and helped preserve reperfusion benefits, even when definitive EVT occurred later or during off-hours. Bridging therapy was consistently associated with higher rates of functional independence (mRS score of 0–2) and lower mortality across both off-hours and business hours, particularly in mothership patients. Although transferred patients showed similar outcomes between groups, bridging remained numerically favorable. Importantly, no significant interaction was found between treatment timing and bridging status, indicating the benefits of IVT remained consistent regardless of presentation time. Although a higher proportion of EVT-alone patients presented during off-hours, this was not statistically significant. Bridging therapy was more common among transferred patients, suggesting that IVT was frequently initiated at referring centers, potentially preserving its benefits, despite transfer-related delays. This supports the continued use of IVT before EVT across different scenarios. In addition, newer thrombolytics, such as TNK (tenecteplase), may further optimize care by simplifying administration and potentially reducing delays compared with alteplase, especially in time-sensitive scenarios.^42^

Safety profiles were comparable between the IVT+EVT and EVT-alone groups, with no significant differences in rates of sICH. This supports the safety of combining IVT with EVT in patients with M2 occlusions, consistent with findings from the HERMES meta-analysis and STRATIS Registry.^10,11^ These results are particularly reassuring given the concern over increased bleeding risk with dual therapy. Our study has important clinical implications for the management of M2-MCA. It reinforces the role of IVT as a complementary therapy to EVT, including patients with M2 MCA occlusions presenting directly to EVT-capable centers. In addition, it highlights the critical need for streamlined prehospital pathways to reduce delays in reperfusion therapy, especially for transfer patients. Future efforts should focus on improving triage systems, reducing door-in-door-out times, and refining patient selection criteria for bridging therapy in transfer scenarios.^43,44^

All patients in our analysis who received IVT were treated with alteplase, consistent with standard practice during the MR CLEAN Registry period. TNK was not in routine use at that time.^24^ However, TNK has since gained interest as an alternative to alteplase due to its greater fibrin specificity, longer half-life, and ease of administration via a single bolus. Although our data set did not include TNK-treated patients, recent randomized data have investigated its potential benefit in the context of bridging therapy. In BRIDGE-TNK trial (Intravenous Tenecteplase Before Thrombectomy in Stroke) comparing TNK plus thrombectomy (n=278) to thrombectomy alone (n=272), functional independence at 90 days was achieved in 52.9% of patients receiving TNK versus 44.1% in the thrombectomy-alone group (unadjusted risk ratio, 1.20 [95% CI, 1.01–1.43]; *P*=0.04). Early reperfusion before thrombectomy was more frequent in the TNK group (6.1% versus 1.1%), while successful reperfusion after thrombectomy was similarly high in both groups (91.4% versus 94.1%). Rates of sICH (8.5% versus 6.7%) and 90-day mortality (22.3% versus 19.9%) were also comparable. Notably, the number of patients with M2 occlusions in this trial was small—18 (6.5%) in the TNK group and 20 (7.4%) in the thrombectomy-alone group—limiting the ability to draw definitive conclusions regarding the impact of TNK in this specific subgroup.^42^ Nonetheless, these findings highlight the need for future prospective studies to evaluate whether TNK may offer additional clinical benefit over alteplase in patients with occlusions, such as MCA-M2.

This study is strengthened by several key factors. It included a large sample size, which enhances the power and generalizability of the findings. The methodology for data collection and assessment was highly rigorous, with an independent core laboratory conducting blinded reviews of baseline NCCT and computed tomography angiography, procedural digital subtraction angiography, and follow-up NCCT to ensure consistent and objective evaluation. Furthermore, the study employed robust statistical methods, incorporating appropriate adjustments for potential confounders, which strengthens the validity and reliability of the results. Several limitations should be acknowledged. Most importantly, in this analysis based on a prospectively maintained registry, IVT was administered based on indication or, conversely, withheld in patients with contraindications. Despite the use of advanced statistical techniques such as IPTW to mitigate confounding, the potential for residual bias remains. In addition, the heterogeneity of M2 occlusions, including differences in clot burden and thrombus characteristics (density, volume, and perviousness), collateral circulation, and anatomic location, could also affect outcomes. We could not differentiate between proximal and distal M2 occlusions, which could influence the treatment response and outcomes.^20,45^ However, it is likely that the majority of our cohort had dominant proximal and dominant M2 occlusions. Prior analysis from this registry demonstrated that among 175 M2 occlusions, 124 (71%) were classified as dominant.^25^ Some M2 segments serve as the primary artery supplying a significant portion of MCA territory. Occlusion in these segments can result in severe neurological deficits and large infarctions, yet they are as readily accessible for EVT as M1 segment occlusions. Therefore, patients with clots located in the dominant M2 artery or near the bifurcation may derive comparable benefits from EVT. Imaging characteristics of M2 MCA occlusions could play a critical role in determining the efficacy and safety of EVT, whether performed alone or in combination with IVT.^36^ At the time of data collection, stent retrievers were the predominant first-line approach, limiting variability. Nonetheless, we conducted an exploratory stratified analysis showing numerically better outcomes with bridging IVT across both aspiration and stent retriever/combined groups. Previous analysis from the MR CLEAN Registry has shown that aspiration-first and stent retriever strategies result in comparable functional outcomes across a range of occlusion locations, including M2 segments. Among 2282 patients, 1658 (73%) underwent initial treatment with stent retrievers, whereas 624 (27%) were treated with aspiration as the first-line technique. The study included a range of occlusion sites: 462 (20%) in the ICA, 1349 (59%) in the MCA-M1, and 471 (21%) in the MCA-M2. Functional outcomes at 90 days did not differ between groups (aOR, 1.0 [95% CI, 0.9–1.2]; *P*=0.20). However, aspiration was associated with higher odds of successful reperfusion (aOR, 1.4 [95% CI, 1.1–1.6]), a benefit that was consistent across occlusion locations (*P*=0.60). In addition, aspiration achieved shorter median procedure times (50 minutes versus 65 minutes; *P*<0.0001). There were no significant differences in the rates of periprocedural complications or mortality between the 2 techniques. These findings suggest that aspiration may offer procedural advantages while maintaining safety and efficacy. Future prospective studies are warranted to assess whether the impact of bridging therapy varies by thrombectomy strategy in contemporary practice.^46^ Moreover, we must acknowledge that we did not have access to patients who responded to IVT and did not require EVT, which could have resulted in a selection bias.^21,39^ Specifically, among transfer patients, those who responded to IVT and did not need EVT were excluded, leaving only IVT-refractory cases, which may skew the results and explain the appearance of limited value of adding IVT in this transferred subgroup. Future studies should explore these variables in greater depth to better inform treatment strategies.

## Conclusions

Our findings suggest that bridging therapy (IVT+EVT) offers superior functional outcomes compared with EVT alone in patients with M2 MCA occlusions. These results emphasize the importance of individualized treatment strategies that consider both patient-level and system-level factors. Further prospective, randomized studies are warranted to confirm these findings and refine treatment protocols, optimizing care for this complex patient population.

## Article Information

### Acknowledgments

The authors thank all the investigators of the MR CLEAN Registry (Multicenter Randomized Controlled Trial of Endovascular Treatment for Acute Ischemic Stroke in the Netherlands) for their effort and contributions.

### Sources of Funding

The MR CLEAN Registry (Multicenter Randomized Clinical Trial of Endovascular Treatment of Acute Ischemic Stroke) was partly funded by Stichting Toegepast Wetenschappelijk Instituut voor Neuromodulatie, Erasmus University Medical Center, Maastricht University Medical Center, and Amsterdam University Medical Center.

### Disclosures

Dr Staals reports compensation from Medtronic for other services. Dr Majoie reports stock holdings in Nico-lab; grants from Health Evaluation the Netherlands to other; grants from Toegepast Wetenschappelijk Instituut voor Neuromodulatie (TWIN) Foundation to other; grants from Boehringer Ingelheim to other; grants from Dutch Heart Foundation to other; grants from Stryker Corporation to other; and grants from European Commission to other. Dr Nogueira reports stock options in Reist/Q’Apel Medical; compensation from Shanghai Wallaby for consultant services; stock holdings in Quantanosis AI; compensation from Corindus Inc for consultant services; stock holdings in Piraeus Medical; compensation from Perfuze for consultant services; compensation from Vesalio for consultant services; compensation from Philips for consultant services; compensation from Brainomix for consultant services; compensation from RapidPulse for consultant services; stock options in viz-AI; compensation from Biogen Inc for consultant services; stock options in Viseon Inc; compensation from Anaconda Biomed for consultant services; compensation from phenox Inc for consultant services; grants from Cerenovus; compensation from NeuroVasc Technologies Inc for consultant services; compensation from Synchron for data and safety monitoring services; stock options in Corindus Inc; compensation from Hybernia for consultant services; compensation from Ceretrieve for consultant services; compensation from Cerenovus for consultant services; compensation from Imperative Care for consultant services; compensation from Genentech for consultant services; compensation from Medtronic USA Inc for consultant services; compensation from Imperative Care Inc for consultant services; stock options in Truvic; stock options in RapidPulse; stock holdings in Brain4Care; grants from Stryker; compensation from Prolong Pharmaceuticals for consultant services; stock options in Brainomix; stock options in Perfuze; stock options in Ceretrieve; compensation from Cerebrotech for consultant services; stock options in Cerebrotech; compensation from Boehringer Ingelheim for consultant services; compensation from viz-AI for consultant services; stock options in Vesalio; compensation from Corindus Vascular Robotics for consultant services; compensation from Stryker Corporation for consultant services; and compensation from Astrocyte for consultant services. Dr van Zwam reports grants from Johnson and Johnson International; grants from Bayer HealthCare Pharmaceuticals Inc; grants from Stryker Corporation; compensation from Philips for data and safety monitoring services; and employment by Maastricht Universitair Medisch Centrum. The other authors report no conflicts.

### Supplemental Material

Tables S1 and S2

STROBE Checklist

## Appendix

MR CLEAN Registry Investigators

Executive Committee

Diederik W.J. Dippel (Department of Neurology, Erasmus University Medical Center), Aad van der Lugt (Department of Radiology, Erasmus University Medical Center), Charles B.L.M. Majoie (Department of Radiology and Nuclear Medicine, Amsterdam UMC, University of Amsterdam), Yvo B.W.E.M. Roos (Department of Neurology, Amsterdam UMC, University of Amsterdam, Amsterdam), Robert J. van Oostenbrugge (Department of Neurology, Maastricht University Medical Center and Cardiovascular Research Institute Maastricht (CARIM), Wim H. van Zwam (Department of Radiology, Maastricht University Medical Center and Cardiovascular Research Institute Maastricht [CARIM]), Jelis Boiten (Department of Neurology, Haaglanden MC, the Hague), Jan Albert Vos (Department of Radiology, St. Antonius Hospital, Nieuwegein).

Study Coordinators

Ivo G.H. Jansen (Department of Radiology and Nuclear Medicine, Amsterdam UMC, University of Amsterdam), Maxim J.H.L. Mulder (Departments of Neurology and Radiology, Erasmus University Medical Center). Robert-Jan B. Goldhoorn (Department of Neurology, and Department of Radiology, Maastricht University Medical Center and Cardiovascular Research Institute Maastricht (CARIM), Kars C.J. Compagne (Department of Radiology, Erasmus University Medical Center), Manon Kappelhof (Department of Radiology and Nuclear Medicine, Amsterdam UMC, University of Amsterdam, Amsterdam), Josje Brouwer (Department of Neurology, Amsterdam UMC, University of Amsterdam, Amsterdam Sanne J. den Hartog (Departments of Neurology, Radiology, and Public Health, Erasmus University Medical Center), Wouter H. Hinsenveld (Departments of Neurology and Radiology, Maastricht University Medical Center and Cardiovascular Research Institute Maastricht (CARIM).

Local Principal Investigators

Diederik W.J. Dippel (Department of Neurology, Erasmus University Medical Center), Bob Roozenbeek (Department of Neurology, Erasmus University Medical Center), Aad van der Lugt (Department of Radiology, Erasmus University Medical Center), Charles B.L.M. Majoie (Department of Radiology and Nuclear Medicine, Amsterdam UMC, University of Amsterdam, Amsterdam), Yvo B.W.E.M. Roos (Department of Neurology, Amsterdam UMC, University of Amsterdam, Amsterdam), Bart J. Emmer (Department of Radiology and Nuclear Medicine, Amsterdam UMC, University of Amsterdam, Amsterdam), Jonathan M. Coutinho (Department of Neurology, Amsterdam UMC, University of Amsterdam, Amsterdam), Wouter J. Schonewille (Department of Neurology, St. Antonius Hospital, Nieuwegein), Jan Albert Vos (Department of Radiology, St. Antonius Hospital, Nieuwegein), Marieke J.H. Wermer (Department of Neurology, Leiden University Medical Center), Marianne A.A. van Walderveen (Department of Radiology, Leiden University Medical Center), Adriaan C.G.M. van Es (Department of Radiology, Leiden University Medical Center), Julie Staals (Department of Neurology, Maastricht University Medical Center and Cardiovascular Research Institute Maastricht [CARIM]), Robert J. van Oostenbrugge (Department of Neurology, Maastricht University Medical Center and Cardiovascular Research Institute Maastricht [CARIM]), Wim H. van Zwam (Department of Radiology, Maastricht University Medical Center and Cardiovascular Research Institute Maastricht [CARIM]), Jeannette Hofmeijer (Department of Neurology, Rijnstate Hospital, Arnhem), Jasper M. Martens (Department of Radiology, Rijnstate Hospital, Arnhem), Geert J. Lycklama à Nijeholt (Department of Radiology, Haaglanden MC, the Hague), Jelis Boiten (Department of Neurology, Haaglanden MC, the Hague), Sebastiaan F. de Bruijn (Department of Neurology, HAGA Hospital, the Hague), Lukas C. van Dijk (Department of Radiology, HAGA Hospital, the Hague), H. Bart van der Worp (Department of Neurology, University Medical Center Utrecht), Rob H. Lo (Department of Radiology, University Medical Center Utrecht), Ewoud J. van Dijk (Department of Neurology, Radboud University Medical Center, Nijmegen), Hieronymus D. Boogaarts (Department of Neurosurgery, Radboud University Medical Center, Nijmegen), J. de Vries (Department of Neurology, Isala Klinieken, Zwolle), Paul L.M. de Kort (Department of Neurology, Elisabeth-TweeSteden ziekenhuis, Tilburg), Julia van Tuijl (Department of Neurology, Elisabeth-TweeSteden ziekenhuis, Tilburg), Jo P. Peluso (Department of Radiology, Elisabeth-TweeSteden ziekenhuis, Tilburg), Puck Fransen (Department of Neurology, Isala Klinieken, Zwolle), Jan S.P. van den Berg (Department of Neurology, Isala Klinieken, Zwolle), Boudewijn A.A.M. van Hasselt (Department of Radiology, Isala Klinieken, Zwolle), Leo A.M. Aerden (Department of Neurology, Reinier de Graaf Gasthuis, Delft), René J. Dallinga (Department of Radiology, Reinier de Graaf Gasthuis, Delft), Maarten Uyttenboogaart (Department of Neurology, University Medical Center Groningen), Omid Eschgi (Department of Radiology, University Medical Center Groningen), Reinoud P.H. Bokkers (Department of Radiology, University Medical Center Groningen), Tobien H.C.M.L. Schreuder (Department of Neurology, Atrium Medical Center, Heerlen), Roel J.J. Heijboer (Department of Radiology, Atrium Medical Center, Heerlen), Koos Keizer (Department of Neurology, Catharina Hospital, Eindhoven), Lonneke S.F. Yo (Department of Radiology, Catharina Hospital, Eindhoven), Heleen M. den Hertog (Department of Neurology, Isala Klinieken, Zwolle), Emiel J.C. Sturm (Department of Neurology, Medisch Spectrum Twente, Enschede) Paul J.A.M. Brouwers (Department of Neurology, Medisch Spectrum Twente, Enschede).

Imaging Assessment Committee

Charles B.L.M. Majoie (Chair; Department of Radiology and Nuclear Medicine, Amsterdam UMC, University of Amsterdam), Wim H. van Zwam (Department of Radiology, Maastricht University Medical Center and Cardiovascular Research Institute Maastricht [CARIM]), Aad van der Lugt (Department of Radiology, Erasmus University Medical Center), Geert J. Lycklama à Nijeholt (Department of Radiology, Haaglanden MC, the Hague), Marianne A.A. van Walderveen (Department of Radiology, Leiden University Medical Center), Marieke E.S. Sprengers (Department of Radiology and Nuclear Medicine, Amsterdam UMC, University of Amsterdam) Sjoerd F.M. Jenniskens (Department of Radiology, Radboud University Medical Center, Nijmegen), René van den Berg (Department of Radiology and Nuclear Medicine, Amsterdam UMC, University of Amsterdam), Albert J. Yoo (Department of Radiology, Texas Stroke Institute), Ludo F.M. Beenen (Department of Radiology and Nuclear Medicine, Amsterdam UMC, University of Amsterdam), Alida A. Postma (Department of Radiology, Maastricht University Medical Center and Cardiovascular Research Institute Maastricht [CARIM]), Stefan D. Roosendaal (Department of Radiology and Nuclear Medicine, Amsterdam UMC, University of Amsterdam), Bas F.W. van der Kallen (Department of Radiology, Haaglanden MC, the Hague), Ido R. van den Wijngaard (Department of Radiology, Haaglanden MC, the Hague), Adriaan C.G.M. van Es (Department of Radiology, Leiden University Medical Center), Bart J. Emmer (Department of Radiology and Nuclear Medicine, Amsterdam UMC, University of Amsterdam), Jasper M. Martens (Department of Radiology, Rijnstate Hospital, Arnhem), Lonneke S.F. Yo (Department of Radiology, Catharina Hospital, Eindhoven), Jan Albert Vos (Department of Radiology, St. Antonius Hospital, Nieuwegein), Joost Bot (Department of Radiology, Amsterdam UMC, Vrije Universiteit van Amsterdam, Amsterdam), Pieter-Jan van Doormaal (Department of Radiology, Erasmus University Medical Center), Anton Meijer (Department of Radiology, Radboud University Medical Center, Nijmegen), Elyas Ghariq (Department of Radiology, Haaglanden MC, the Hague), Reinoud P.H. Bokkers (Department of Radiology, University Medical Center Groningen), Marc P. van Proosdij (Department of Radiology, Noordwest Ziekenhuisgroep, Alkmaar), G. Menno Krietemeijer (Department of Radiology, Catharina Hospital, Eindhoven), Jo P. Peluso (Department of Radiology, Elisabeth-TweeSteden ziekenhuis, Tilburg), Hieronymus D. Boogaarts (Department of Neurosurgery, Radboud University Medical Center, Nijmegen), Rob Lo (Department of Radiology, University Medical Center Utrecht), Wouter Dinkelaar (Department of Radiology, Albert Schweitzer Hospital, Dordrecht), Auke P.A. Appelman (Department of Radiology, University Medical Center Groningen), Bas Hammer (Department of Radiology, HAGA Hospital, the Hague), Sjoert Pegge (Department of Neurology Radiology, Radboud University Medical Center, Nijmegen), Anouk van der Hoorn (Department of Radiology, University Medical Center Groningen), Saman Vinke (Department of Neurosurgery, Radboud University Medical Center, Nijmegen), Sandra Cornelissen (Department of Radiology, Erasmus University Medical Center), Christiaan van der Leij (Department of Radiology, Maastricht University Medical Center and Cardiovascular Research Institute Maastricht [CARIM]), Rutger Brans (Department of Radiology, Maastricht University Medical Center and Cardiovascular Research Institute Maastricht [CARIM]).

Writing Committee

Diederik W.J. Dippel (chair; Department of Neurology, Erasmus University Medical Center), Aad van der Lugt (Department of Radiology, Erasmus University Medical Center), Charles B.L.M. Majoie (Department of Radiology and Nuclear Medicine, Amsterdam UMC, University of Amsterdam), Yvo B.W.E.M. Roos (Department of Neurology, Amsterdam UMC, University of Amsterdam), Robert J. van Oostenbrugge (Department of Neurology, Maastricht University Medical Center and Cardiovascular Research Institute Maastricht [CARIM]), Wim H. van Zwam (Department of Radiology, Maastricht University Medical Center and Cardiovascular Research Institute Maastricht [CARIM]), Geert J. Lycklama à Nijeholt (Department of Radiology, Haaglanden MC, the Hague), Jelis Boiten (Department of Neurology, Haaglanden MC, the Hague), Jan Albert Vos (Department of Radiology, St. Antonius Hospital, Nieuwegein), Wouter J. Schonewille (Department of Neurology, St. Antonius Hospital, Nieuwegein), Jeannette Hofmeijer (Department of Neurology, Rijnstate Hospital, Arnhem), Jasper M. Martens (Department of Radiology, Rijnstate Hospital, Arnhem), H. Bart van der Worp (Department of Neurology, University Medical Center Utrecht), Rob H. Lo (Department of Radiology, University Medical Center Utrecht).

Adverse Event Committee

Robert J. van Oostenbrugge (chair; Department of Neurology, Maastricht University Medical Center and Cardiovascular Research Institute Maastricht [CARIM]), Jeannette Hofmeijer (Department of Radiology, Rijnstate Hospital, Arnhem), H. Zwenneke Flach (Department of Radiology, Isala Klinieken, Zwolle).

Trial Methodologist

Hester F. Lingsma (Department of Public Health, Erasmus University Medical Center).

Research Nurses/Local Trial Coordinators

Naziha el Ghannouti (Department of Neurology, Erasmus University Medical Center), Martin Sterrenberg (Department of Neurology, Erasmus University Medical Center), Wilma Pellikaan (Department of Neurology, St. Antonius Hospital, Nieuwegein), Rita Sprengers (Department of Neurology, Amsterdam UMC, University of Amsterdam), Marjan Elfrink (Department of Neurology, Rijnstate Hospital, Arnhem), Michelle Simons (Department of Neurology, Rijnstate Hospital, Arnhem), Marjolein Vossers (Department of Radiology, Rijnstate Hospital, Arnhem), Joke de Meris (Department of Neurology, Haaglanden MC, the Hague), Tamara Vermeulen (Department of Neurology, Haaglanden MC, the Hague), Annet Geerlings (Department of Neurology, Radboud University Medical Center, Nijmegen), Gina van Vemde (Department of Neurology, Isala Klinieken, Zwolle), Tiny Simons (Department of Neurology, Atrium Medical Center, Heerlen), Gert Messchendorp (Department of Neurology, University Medical Center Groningen), Nynke Nicolaij (Department of Neurology, University Medical Center Groningen), Hester Bongenaar (Department of Neurology, Catharina Hospital, Eindhoven), Karin Bodde Department of Neurology, Reinier de Graaf Gasthuis, Delft Sandra Kleijn (Department of Neurology, Medisch Spectrum Twente, Enschede), Jasmijn Lodico (Department of Neurology, Medisch Spectrum Twente, Enschede), Hanneke Droste (Department of Neurology, Medisch Spectrum Twente, Enschede), Maureen Wollaert (Department of Neurology, Maastricht University Medical Center and Cardiovascular Research Institute Maastricht [CARIM]), Sabrina Verheesen (Department of Neurology, Maastricht University Medical Center and Cardiovascular Research Institute Maastricht [CARIM]), D. Jeurrissen (Department of Neurology, Maastricht University Medical Center and Cardiovascular Research Institute Maastricht [CARIM]), Erna Bos (Department of Neurology, Leiden University Medical Center), Yvonne Drabbe (Department of Neurology, HAGA Hospital, the Hague), Michelle Sandiman (Department of Neurology, HAGA Hospital, the Hague), Nicoline Aaldering (Department of Neurology, Rijnstate Hospital, Arnhem), Berber Zweedijk (Department of Neurology, University Medical Center Utrecht), Jocova Vervoort (Department of Neurology, Elisabeth-TweeSteden ziekenhuis, Tilburg), Eva Ponjee (Department of Neurology, Isala Klinieken, Zwolle) Sharon Romviel (Department of Neurology, Radboud University Medical Center, Nijmegen), Karin Kanselaar (Department of Neurology, Radboud University Medical Center, Nijmegen), Denn Barning (Department of Radiology, Leiden University Medical Center),

Clinical/Imaging Data Acquisition

Esmee Venema (Department of Public Health, Erasmus University Medical Center) Vicky Chalos (Departments of Neurology and Public Health, Erasmus University Medical Center) Ralph R. Geuskens (Department of Radiology and Nuclear Medicine, Amsterdam UMC, University of Amsterdam), Tim van Straaten (Department of Neurology, Radboud University Medical Center, Nijmegen), Saliha Ergezen (Department of Neurology, Erasmus University Medical Center), Roger R.M. Harmsma (Department of Neurology, Erasmus University Medical Center), Daan Muijres (Department of Neurology, Erasmus University Medical Center) Anouk de Jong (Department of Neurology, Erasmus University Medical Center), Olvert A. Berkhemer (Department of Neurology, Erasmus University Medical Center; Department of Radiology and Nuclear Medicine, Amsterdam UMC, University of Amsterdam; Department of Radiology, Maastricht University Medical Center and Cardiovascular Research Institute Maastricht [CARIM]), Anna M.M. Boers (Department of Radiology and Nuclear Medicine, Amsterdam UMC, University of Amsterdam) J. Huguet (Department of Radiology and Nuclear Medicine, Amsterdam UMC, University of Amsterdam), P.F.C. Groot (Department of Radiology and Nuclear Medicine, Amsterdam UMC, University of Amsterdam), Marieke A. Mens (Department of Radiology and Nuclear Medicine, Amsterdam UMC, University of Amsterdam), Katinka R. van Kranendonk (Department of Radiology and Nuclear Medicine, Amsterdam UMC, University of Amsterdam), Kilian M. Treurniet (Department of Radiology and Nuclear Medicine, Amsterdam UMC, University of Amsterdam), Manon L. Tolhuisen (Department of Radiology and Nuclear Medicine, Biomedical Engineering & Physics, Amsterdam UMC, University of Amsterdam), Heitor Alves (Department of Radiology and Nuclear Medicine, Amsterdam UMC, University of Amsterdam), Annick J. Weterings (Department of Radiology and Nuclear Medicine, Amsterdam UMC, University of Amsterdam), Eleonora L.F. Kirkels (Department of Radiology and Nuclear Medicine, Amsterdam UMC, University of Amsterdam), Eva J.H.F. Voogd (Department of Neurology, Rijnstate Hospital, Arnhem), Lieve M. Schupp (Department of Radiology and Nuclear Medicine, Amsterdam UMC, University of Amsterdam), Sabine L. Collette (Departments of Neurology and Radiology, University Medical Center Groningen), Adrien E.D. Groot (Department of Neurology, Amsterdam UMC, University of Amsterdam, Amsterdam), Natalie E. LeCouffe (Department of Neurology, Amsterdam UMC, University of Amsterdam, Amsterdam), Praneeta R. Konduri (Department of Biomedical Engineering & Physics, Amsterdam UMC, University of Amsterdam), Haryadi Prasetya (Department of Biomedical Engineering & Physics, Amsterdam UMC, University of Amsterdam), Nerea Arrarte-Terreros (Department of Biomedical Engineering & Physics, Amsterdam UMC, University of Amsterdam), Lucas A. Ramos (Department of Biomedical Engineering & Physics, Amsterdam UMC, University of Amsterdam), Nikki Boodt (Departments of Neurology, Radiology, and Public Health, Erasmus University Medical Center), F. Anne V. Pirson (Department of Neurology, Maastricht University Medical Center and Cardiovascular Research Institute Maastricht [CARIM]), Agnetha A.E. Bruggeman (Department of Radiology and Nuclear Medicine, Amsterdam UMC, University of Amsterdam).

## Supplementary Material


