# Supplementary material for: Direct Endovascular Versus Bridging Therapy in M2 Segment Occlusion of Middle Cerebral Artery: A MR CLEAN Registry Study
Source: Stroke. 2025 Jul 22;56(10):2866–78. doi: 10.1161/STROKEAHA.125.051967 (PMC12447822; doi:10.1161/STROKEAHA.125.051967)
Supplement: Supplementary file 1 [file str-56-2866-s001.pdf]

SUPPLEMENTAL MATERIAL

Table S1: mRS Distribution at 90 days and Key Outcomes by EVT Technique and Bridging Status

EVT Technique	Bridging Status	mRS 0-1 N (%)	mRS 0-2 N (%)	mRS 0-3 N (%)	Mortality at 90 days N (%)	Total
Stent Retriever or Combined	Non-bridging	35 (30.4%)	54 (47.0%)	67 (58.3%)	31 (27.0%)	115
	Bridging	91 (41.0%)	131 (59.0%)	160 (72.1%)	38 (17.1%)	222
Aspiration	Non-bridging	9 (27.3%)	13 (39.4%)	20 (60.6%)	9 (27.3%)	33
	Bridging	30 (34.1%)	49 (55.7%)	63 (71.6%)	17 (19.3%)	88

Table S2: mRS Distribution at 90 days and Key Outcomes by Hospital Type, Time of Day, and Bridging Status, with Interaction Analysis Results

Patient Group / Time of Day	Bridging Status	mRS 0-1 N (%)	mRS 0-2 N (%)	mRS 0-3 N (%)	Mortality N (%)	Total
Overall Cohort						
Off-hours	Non-bridging	31 (30.10%)	48 (46.60%)	62 (60.19%)	27 (26.21%)	103
	Bridging	71 (36.60%)	106 (54.64%)	143 (73.71%)	29 (14.95%)	194
Business Hours	Non-bridging	15 (28.85%)	24 (46.15%)	30 (57.69%)	13 (25.00%)	52
	Bridging	58 (42.03%)	86 (62.32%)	100 (72.46%)	28 (20.29%)	138
Transferred Patients						
Off-hours	Non-bridging	8 (29.63%)	15 (55.56%)	19 (70.37%)	4 (14.81%)	27
	Bridging	33 (36.66%)	49 (54.44%)	67 (74.44%)	15 (16.67%)	90
Business Hours	Non-bridging	3 (37.50%)	4 (50.00%)	5 (62.50%)	2 (25.00%)	8
	Bridging	27 (43.55%)	37 (59.68%)	43 (69.36%)	14 (22.58%)	62
Mothership Patients						
Off-hours	Non-bridging	23 (30.26%)	33 (43.42%)	43 (56.58%)	23 (30.26%)	76
	Bridging	38 (36.54%)	57 (54.81%)	76 (73.08%)	14 (13.46%)	104
Business Hours	Non-bridging	12 (27.28%)	20 (45.46%)	25 (56.82%)	11 (25.00%)	44
	Bridging	31 (40.79%)	49 (64.47%)	57 (75.00%)	14 (18.42%)	76
